# The positive influence the Onchocerciasis Elimination Program for the Americas has had on Africa programs

**DOI:** 10.1186/s40249-019-0558-0

**Published:** 2019-07-15

**Authors:** Frank O. Richards, B. E. B. Nwoke, Isam Zarroug, Edridah Tukahebwa, Nebiyu Negussu, T. B. Higazi, David Oguttu, Zerihun Tadesse, Emmanuel Miri, Nabil Aziz, Peace Habomugisha, Moses Katabarwa

**Affiliations:** 1 The Carter Center, One Copenhill Avenue, 453 Freedom Parkway, Atlanta, GA 30307 USA; 2 Imo State University Owerri, PMB 2000, Owerri, Nigeria; 3 Sudan Ministry of Health, P.O. Box 303, Khartoum, Sudan; 4 Uganda Ministry of Health, 15 Bombo Rd., P. O. Box 1661, Kampala, Uganda; 5 Ethiopia Federal Ministry of Health, P. O. Box 1234 Addis Ababa, Ethiopia; 6 Ohio University, 1425 Newark Rd, Zanesville, OH 43701 USA; 7 The Carter Center-Ethiopia, P. O. Box 13373, Bole K. K. Kebele 05, Addis Ababa, Ethiopia; 8 The Carter Center-Nigeria, No. 1 Jeka Kadima St, Jos, Plateau State Nigeria; 9 The Carter Center-Sudan, P. O. Box 48, Khartoum, Sudan; 10 The Carter Center-Uganda, P.O. Box 12027, Kampala, Uganda

**Keywords:** Onchocerciasis, Elimination, Ivermectin mass drug administration, Onchocerciasis control Programme for West Africa, Test for antibody to *Onchocerca volvulus* 16 kDa antigen

## Abstract

A recent article “Is onchocerciasis elimination in Africa feasible by 2025: a perspective based on lessons learnt from the African control programmes” in *Infectious Diseases of Poverty* claimed that undue influence on African programs by concepts developed by the Onchocerciasis Elimination Program of the Americas (OEPA) is detrimental to stopping mass drug administration (MDA) in Africa. This claim is made despite a record year for MDA stoppage in four African countries of > 3.5 million treatments in 2018, far exceeding any past OEPA or African Program for Onchocerciasis Control (APOC) stop MDA success.

**Electronic supplementary material:**

The online version of this article (10.1186/s40249-019-0558-0) contains supplementary material, which is available to authorized users.

## Multilingual abstracts

Please see Additional file 1 for translations of the abstract into the five official working languages of the United Nations.

## Background

Professor Dadzie with other distinguished directors of the former World Health Organization (WHO) African Regional Programs against River Blindness (the Onchocerciasis Control Programme in West Africa [OCP] and the African Programme for Onchocerciasis Control [APOC]) recently published an article in *Infectious Diseases of Poverty* including the claims: (1) that the influence of the ongoing Onchocerciasis Elimination Program of the Americas (OEPA) is, “impeding progress towards decisions to stop intervention in many (African) areas that have reached the elimination point” and (2) that the introduction of testing for antibody to the *Onchocerca volvulus* 16 kDa antigen (OV16) in children (operationalized by OEPA) to assess for evidence of recent transmission “has delayed progress with stopping treatment which according to APOC evaluations should already be feasible for millions of people.” [1] We write to respectfully contest those two assertions.

## Main text

The OEPA programmatic model consists of five basic principles [2, 3]: (1) Interrupt onchocerciasis transmission through aggressive, enhanced and flexible interventions, and most especially by six monthly ivermectin mass drug administration (MDA) in all communities where transmission exists. (2) Use the WHO Geneva guidelines (first published in 2001, later revised in 2016) in an elimination paradigm consisting of three milestones [4, 5]: (i) *transmission suppression*, at which point the adult *Onchocerca volvulus* worm population is in demise; (ii) *transmission interruption* determined by epidemiological and entomological studies of the *Simulium* vector black flies, at which time MDA may be halted; and lastly (iii) *Post Treatment Surveillance* (PTS) for 3–5 years, after which evaluations must successfully demonstrate lack of recrudescence, at which time *transmission elimination* may be declared. (3) Decisions to stop MDA and PTS based on these WHO Guidelines require transmission monitoring in children. On this point, it should be recognized that it was the WHO Geneva 2001 guidelines (not OEPA) that stipulated what we agree is a quite challenging 0.1% threshold to measure [4, 6, 7]. This threshold however was retained by WHO in its 2016 guideline revision after a thorough review by methodologists [5], at which time WHO called for the use of OV16 antibody testing instead of insensitive and unpopular skin snips [8–10]. Both 2001 and 2016 WHO guidelines call for Polymerase Chain Reaction (PCR) amplification of the *O. volvulus* 150 bp tandem repeat (O150) in pools of black fly heads (the threshold being < 1/2000 infective flies) rather than dissection. Because a laboratory was required for the PCR, in the mid-2000s OEPA successfully operationalized the use of OV16 antibody monitoring by Enzyme Linked Immunosorbant Assay (ELISA) testing of dried blood spots since it could be performed in the same facility. This test is currently known as the ‘OEPA’ OV16 ELISA [6]. National labs were established with OEPA support whenever possible and the University of South Florida, now a WHO reference lab for onchocerciasis diagnostics, provided technical oversight. (4) OEPA promoted national program ownership and responsibility by encouraging the programs to decide for themselves how to best deliver ivermectin within their individual health systems. National programs were supported by a regional OEPA committee called the Program Coordinating Committee (PCC). The PCC includes WHO representation and provides recommendations and offers technical/financial assistance when needed. However, all decisions for subnational and national action were made solely by the countries themselves, and not by the regional committee. (5) Where active onchocerciasis transmission spanned international borders, OEPA together with the regional WHO office worked with authorities on both sides to establish ‘Special Intervention Zones’ (SIZs) (a term borrowed from OCP) to help with the inevitable political challenges facing the programs [3, 11]. Under this paradigm about 95% of the MDA for onchocerciasis in the Americas has been halted [3].

This five-step OEPA model had a positive influence in Africa due to two annual meetings that provided a forum for onchocerciasis warriors from six African countries (Cameroon, Uganda, Nigeria, Ethiopia, Sudan, and South Sudan) and six American ones (Colombia, Ecuador, Mexico, Guatemala, Brazil, and Venezuela) to exchange information. One of these meetings was the annual InterAmerican Conferences for Onchocerciasis (IACO, held since 1992), and the other was the annual Carter Center Program Review (held since 1996). On several occasions African country delegations attended IACO and at least one IACO an APOC Director (Dr. A. Seketeli) gave the keynote address. The Director of OEPA (Dr. M. Sauerbrey) attended at least one meeting of the APOC Technical Consultative Committee (TCC).

The fruit of this African-American exchange first became apparent in 2006 when Sudan declared elimination of transmission of onchocerciasis as its goal. In doing so, Sudan embraced the 2001 WHO Geneva Elimination Guidelines and many OEPA principles to reorient its program. The national program independently took the decision to upgrade to OEPA’s twice-per-year treatment strategy and successfully adapted it to the APOC Community Directed Treatment with Ivermectin (CDTI) framework. It expanded MDA to low prevalence (hypoendemic) communities included under OEPA but excluded under the APOC paradigm. A national lab was established at the Ministry of Health, and OEPA OV16 ELISA and O150 PCR were successfully deployed to allow a stop MDA decision in Abu Hamad in 2012 and completion of 3-year PTS [12, 13]. The Abu Hamad focus became the first in Africa to eliminate onchocerciasis transmission outside of a research setting, and it was the first to do so under WHO Geneva Guidelines using the OV16 threshold. The Sudanese publications cite OEPA as an inspiration to move from annual to twice-per-year MDA and present data to support that the change in national policy resulted in elimination [12].

In 2007, Uganda declared a goal of onchocerciasis transmission elimination from all its 16 active transmission zones (foci) just months after a high-level delegation went to Guatemala for the 2006 IACO. The 2018 publication describing the history of the Uganda program includes in its introduction a section entitled ‘Inspiration from the Americas’ [14]. The Uganda Onchocerciasis Elimination Expert Advisory Committee (UOEEAC) was modeled on the OEPA PCC. The first UOEEAC was held in 2008, together with the launching of a national twice-per-year treatment policy, establishment of a molecular lab at the Ministry of Health to support OEPA OV16 ELISA and O150 PCR testing, and deployment of vector elimination/control through ground larviciding in most of Uganda’s *Simulium neavei* foci. The UOEEAC defined guidelines for elimination in *S. neavei* areas that were ultimately incorporated into the 2016 WHO guidelines. Representatives from onchocerciasis programs in the Democratic Republic of Congo and the Republic of South Sudan regularly attend UOEEAC meetings to discuss establishing SIZs in shared (cross-border) transmission zones with Uganda. Since the Ugandan program launched its elimination policy, approximately 1.9 million ivermectin treatments have been halted in the country. Active transmission now only occurs in two of the original foci. The *S. neavei* vector has been eliminated from many foci. Six foci have been determined to have met the WHO criteria for elimination by successfully completing the 3-year PTS period; an estimated 1.15 million persons living in these districts are no longer at risk of acquiring onchocerciasis [15]. To our knowledge, this is the largest national population ever declared free of onchocerciasis. Uganda is widely considered to be the model program of the African onchocerciasis elimination effort.

Ethiopia is now in its sixth year of executing a national twice-per-year treatment policy to accelerate onchocerciasis transmission elimination. In 2017, at its third meeting, the Ethiopian Onchocerciasis Elimination Expert Advisory Committee (EOEEAC) met with representatives from the Sudan program to review binational PCR and OV16 data [16]. The analysis resulted in a joint declaration to stop ivermectin MDA in a cross-border SIZ connecting eight districts of the North Gondar zone of the Amhara region in Ethiopia with the Galabat district of Sudan’s Gedaref state [15–18]. Over 1 million treatments were stopped in a coordinated binational fashion in 2018. The Ethiopian PCR and OV16 testing was conducted at the Ethiopian Public Health Institute (EPHI). OEPA OV16 ELISA is being used as the diagnostic for mapping in the east of the country, and in 2018 a putative unrecognized focus of onchocerciasis was discovered in eastern Oromia Region. Confirmatory investigations of this area are planned.

At its fifth meeting, the Nigeria Onchocerciasis Elimination Committee (NOEC) reviewed results of 2017 OV16/PCR assessments in Plateau and Nasarawa States and determined that the WHO Geneva guidelines for stopping ivermectin MDA had been met [19]. It recommended to the Federal Ministry of Health (FMOH) that MDA be halted there after 24 years of annual MDA. The FMOH accepted the recommendation and stopped 2.6 million treatments in 2018, the largest single stop MDA for onchocerciasis ever [19]. The testing that supported this decision was conducted in a lab based at The Carter Center headquarters in Jos, Nigeria. This same lab completed testing of specimens from Kaduna state where NOEC recommended that MDA could be stopped in 2019. This will be another record MDA stoppage. Of particular interest is that seven years ago Tekle et al. 2012 reported Kaduna’s progress toward onchocerciasis elimination, yet noted MDA had to continue there [20]. The delay in Kaduna was not due to failure to reach the 0.1% OV16 serology threshold, but to the challenge of collecting the 6000 vector black flies for PCR testing required by both APOC and WHO Geneva elimination guidelines [4, 5, 21]. It is therefore quite interesting that in 2018 the Kaduna state onchocerciasis program finally obtained the requisite 6000 fly collection after the NOEC approved and encouraged the use of the Esperanza fly trap to supplement human landing captures. The Esperanza fly trap was first developed with OEPA support in Mexico [22].

## Conclusions

We conclude by noting that 2018 was the most successful year ever for stopping MDA for onchocerciasis in Africa, and that the OEPA elimination model was important in helping that success. Last year 3.8 million ivermectin treatments for onchocerciasis were halted in Africa, 64% of the cumulative 5.9 million treatments that have been stopped since Sudan’s initial Abu Hamad success in 2012. This is compared with under 1 million treatments stopped by OEPA in the Americas [3]. All of these African stop MDA decisions were made by national programs in consultation with their committees and following the WHO Geneva guidelines. All have made their decisions based on data that included OV16 ELISA testing using OEPA methodology [6] and conducted in national labs run by national technicians, without the need for invasive, insensitive and unpopular skin snips [8–10]. These exciting and positive developments should rally the public health community to embrace the opportunity to achieve onchocerciasis transmission elimination in Africa.

## Additional file

Additional file 1: Multilingual abstracts in the five official working languages of the United Nations. (PDF 321 kb)

## Data Availability

Not applicable.

## Abbreviations

APOC: African Programme for Onchocerciasis Control
ELISA: Enzyme Linked Immunosorbant Assay
EOEEAC: Ethiopian Onchocerciasis Elimination Expert Advisory Committee
FMOH: Federal Ministry of Health
IACO: InterAmerican Conferences for Onchocerciasis
MDA: Mass drug administration
NOEC: Nigeria Onchocerciasis Elimination Committee
O150: *O. volvulus* 150 bp tandem repeat
OCP: Onchocerciasis Control Programme for West Africa
OEPA: Onchocerciasis Elimination Program of the Americas
OV16: Test for antibody to *Onchocerca volvulus* 16 kDa antigen
PCC: Program Coordinating Committee of OEPA
PCR: Polymerase Chain Reaction
PTS: Post Treatment Surveillance
SIZ: Special Intervention Zones on international borders
TCC: Technical Consultative Committee of APOC
UOEEAC: Uganda Onchocerciasis Elimination Expert Advisory Committee
WHO: World Health Organization
