# Supplementary material for: The positive influence the Onchocerciasis Elimination Program for the Americas has had on Africa programs
Source: Infect Dis Poverty. 2019 Jul 15;8:52. doi: 10.1186/s40249-019-0558-0 (PMC6628490; doi:10.1186/s40249-019-0558-0)
Supplement: Supplementary file 1 — Multilingual abstracts in the five official working languages of the United Nations. (PDF 321 kb) [file 40249_2019_558_MOESM1_ESM.pdf]

Translation of the abstract into the five official working languages of the United Nations

التأثير الإيجابي على البرامج الأفريقية الناتج من برنامج القضاء على داء كلابية الذنب للأمريكتين

فرانك أو. ريتشاردز،ب.اي.ب نوك، وعصام زروق، وادريه توكيهبوا، ونيبيو نيجوسو،ت. ب. حجازي، وديفيد اوغوتو، وزيريهون تاديسي، وإيمانويل ميري، ونبيل عزيز، والسلام هابوموجيشا، وموسى كتاباروا

مُلخص
ادّعت مقالة حديثة بعنوان "هل القضاء على داء كلابية الذنب في أفريقيا سيكون ممكنًا بحلول عام 2025: منظور قائم على الدروس المستفادة من برامج المكافحة في أفريقيا" في مجلة "أمراض الفقر المعدية" أنَّ التأثير المُفرط على البرامج الأفريقية الناتج من برنامج القضاء على داء كلابية الذنب للأمريكتين (OEPA) مما يضر بإيقاف تقديم العقاقير الشامل (MDA) في أفريقيا، وجاء هذا البيان على الرغم من عام قياسي توقف فيه تقديم العقاقير الشامل في أربعة بلدان أفريقية لأكثر من 3.5 مليون علاج في عام 2018، وهو ما يتجاوز بكثير أي برنامج القضاء على داء كلابية الذنب للأمريكتين أو برنامج مكافحة داء كلابية الذنب الأفريقي الذي أوقف نجاح تقديم الأدوية الشامل.

Translated from English version into Arabic by Irene Campo, Revised by Muhannad al-Bayk, through

TRANSLATORS WITHOUT BORDERS

美洲消除盘尾丝虫病项目对非洲项目产生积极影响

Frank O. Richards, B.E.B Nwoke, Isam Zarroug, Edridah Tukahebwa, Nebiyu Negussu, T. B. Higazi, David Oguttu, Zerihun Tadesse, Emmanuel Miri, Nabil Aziz, Peace Habomugisha, Moses Katabarwa

在《贫困所致传染》上最近一篇文章“2025 年在非洲盘尾丝虫病消除是否可行？基于非洲控制项目经验教训”中，该研究认为美洲消除盘尾丝虫病规划(OEPA)提出的概念对非洲规划产生的不适当影响不利于停止非洲的大规模群体服药(MDA)。但有 4 个非洲国家在 2018 年终止了创纪录的大于 50 万次群体治疗，远远超过了以往任何 OEPA 或非洲控制盘尾丝虫病(APOC)项目阻止 MDA 的记录。

Translated from English version into Chinese by Xin-Yu Feng, , edited by Jin Chen

Influence positive du Programme d’élimination de l’onchocercose pour les Amériques sur les programmes africains

Frank O. Richards, B.E.B Nwoke, Isam Zarroug, Edridah Tukahebwa, Nebiyu Negussu, T. B. Higazi, David Oguttu, Zerihun Tadesse, Emmanuel Miri, Nabil Aziz, Peace Habomugisha, Moses Katabarwa

Résumé

Un récent article intitulé « L’élimination de l’onchocercose en Afrique est-elle réalisable d’ici 2025 : une perspective fondée sur les enseignements tirés des programmes de lutte en Afrique », publié dans *Infectious Diseases of Poverty*, avance que les concepts du Programme pour l’élimination de l’onchocercose pour les Amériques (OEPA) influencent indûment les programmes africains et empêchent de parvenir à l’arrêt des administrations en masse de médicaments (AMD) en Afrique. Cette affirmation va à l’encontre des résultats d’une année record pour les arrêts d’AMD dans quatre pays africains en 2018 (plus de 3,5 millions de traitements), dépassant de loin les succès passés de l’OEPA ou du Programme africain de lutte contre l’onchocercose (APOC).

Translated from English version into French by Marina Della Torre, Revised by Suzanne Assenat, through

TRANSLATORS
WITHOUT BORDERS

Положительное воздействие, которое оказали результаты программы по ликвидации онхоцеркоза в Северной и Южной Америках на программы в Африке

Фрэнк О. Ричардс, Б.Э.Б Нвоук, Исам Зарроуг, Эдрида Тукахебва, Небию Негуссу, Т. Б. Хигази, Дэвид Огутту, Зерихун Тадессе, Эммануэль Мири, Набиль Азиз, Пис Хабомугиша, Мозес Катабарва

Реферат

В недавно опубликованной в журнале *Infectious Diseases of Poverty* статье «Возможна ли ликвидация онхоцеркоза в Африке к 2025 г.: подход, основанный на опыте, полученном из африканских программ по борьбе с болезнью» заявляется о том, что ненадлежащее влияние на африканские программы концепций, разработанных Программой ликвидации онхоцеркоза в Северной и Южной Америках (OEPA), пагубно сказывается на прекращении массового применения лекарственных препаратов в Африке. Это заявление было сделано несмотря на рекордные годовые показатели по прекращению массового введения лекарственных препаратов в четырех африканских странах, что составило > 3,5 миллионов терапий в 2018 г., что значительно превышает результаты любой предыдущей программы OEPA или африканской программы борьбы с онхоцеркозом (АПБО) по прекращению массового применения лекарственных препаратов.

Translated from English version into Russian by Veronika Demeshchik, Revised by Michael Orlov, through

El efecto positivo que ha tenido el Programa para la Eliminación de la Oncocercosis en las Américas en programas para África.

Frank O. Richards, B. E. B. Nwoke, Isam Zarroug, Edridah Tukahebwa, Nebiyu Negussu, T. B. Higazi, David Oguttu, Zerihun Tadesse, Emmanuel Miri, Nabil Aziz, Peace Habomugisha, Moses Katabarwa.

Resumen

El artículo titulado "¿Es viable la eliminación de la Oncocercosis para el año 2025? Una perspectiva basada en las enseñanzas adquiridas por programas de control africanos" recientemente publicado en *Enfermedades Infecciosas de la Pobreza (en inglés, Infectious Diseases of Poverty)* afirmaba que el condicionamiento indebido en programas africanos por conceptos desarrollados por el Programa para la Eliminación de la Oncocercosis en las Américas (OEPA) es perjudicial para la suspensión de la administración masiva de medicamentos (AMM) en África. Esta afirmación fue emitida pese a que el 2018 fue un año récord con la suspensión de la AMM de más de 3,5 millones de tratamientos en cuatro países africanos, sobrepasando con creces a todos los resultados anteriores de suspensiones de AMM de la OEPA o del Programa Africano de Lucha contra la Oncocercosis (APOC).

Translated from English version into Spanish by Maria Gracia Zavarse, Revised by Natalia Victoria Gómez, through
